# Transcriptomic analyses reveal increased expression of dioxygenases, monooxygenases, and other metabolizing enzymes involved in anthracene degradation in the marine alga *Ulva lactuca*

**DOI:** 10.3389/fpls.2022.955601

**Published:** 2022-09-20

**Authors:** Alberto González, Héctor Osorio, Stephanie Romero, Patricia Méndez, Muriel Sepúlveda, Daniel Laporte, Marlen Gutierrez-Cutiño, Rocío Santander, Eduardo Castro-Nallar, Alejandra Moenne

**Affiliations:** ^1^Laboratory of Marine Biotechnology, Faculty of Chemistry and Biology, University of Santiago of Chile, Santiago, Chile; ^2^Laboratorio Multidisciplinario, Instituto de Ciencias Biomédicas, Universidad Autónoma de Chile, Talca, Chile; ^3^Faculty of Chemistry and Biology, University of Santiago of Chile, Santiago, Chile; ^4^Departamento de Microbiología, Facultad de Ciencias de la Salud, Universidad de Talca, Talca, Chile; ^5^Centro de Ecología Integrativa, Universidad de Talca, Talca, Chile

**Keywords:** anthracene, dioxygenase, hydrolases, marine alga, monooxygenases, *Ulva lactuca*

## Abstract

To analyze the mechanisms involved in anthracene (ANT) degradation in the marine alga *Ulva lactuca*, total RNA was obtained from the alga cultivated without ANT and with 5 μM of ANT for 24 h, and transcriptomic analyses were performed. A *de novo* transcriptome was assembled, transcripts differentially expressed were selected, and those overexpressed were identified. Overexpressed transcripts potentially involved in ANT degradation were: one aromatic ring dioxygenase, three 2-oxoglutarate Fe (II) dioxygenases (2-OGDOs), and three dienelactone hydrolases that may account for anthraquinone, phthalic anhydride, salicylic acid, and phthalic acid production (pathway 1). In addition, two flavin adenine dinucleotide (FAD)-dependent monooxygenases, four cytP450 monooxygenases, two epoxide hydrolase, one hydroxyphenylpyruvic acid dioxygenase (HPPDO), and two homogentisic acid dioxygenases (HGDOs) were identified that may also participate in ANT degradation (pathway 2). Moreover, an alkane monooxygenase (alkB), two alcohol dehydrogenases, and three aldehyde dehydrogenases were identified, which may participate in linear hydrocarbon degradation (pathway 3). Furthermore, the level of transcripts encoding some of mentioned enzymes were quantified by qRT-PCR are in the alga cultivated with 5 μM of ANT for 0–48 h, and those more increased were 2-OGDO, HGDO, and alkB monooxygenase. Thus, at least three pathways for ANT and linear hydrocarbons degradation may be existed in *U. lactuca*. In addition, ANT metabolites were analyzed by gas chromatography and mass spectrometry (GC–MS), allowing the identification of anthraquinone, phthalic anhydride, salicylic acid, and phthalic acid, thus validating the pathway 1.

## Introduction

Polycyclic aromatic hydrocarbons (PAHs) are planar aromatic molecules constituted by two to six benzene rings ordered in linear, clustered, or angular arrangements (Mojiri et al., 2019; Patel et al., 2020). In particular, anthracene (ANT) and phenanthrene (PHE) are constituted by three aromatic rings, pyrene (PYR) by four aromatic rings and benzo[a]pyrene (BaP) by five aromatic rings (Mojiri et al., 2019; Patel et al., 2020). PAHs are present in petroleum and diesel, and they are also produced by incomplete combustion of wood, oils, and coal. PAHs are resistant to biodegradation, and they are accumulated in water, sediments, and soils (Mojiri et al., 2019; Patel et al., 2020). PAHs are hydrophobic molecules that can cross the cellular membrane and accumulate into the cells, mainly in organelles (Gao et al., 2013). PAHs are harmful to cell membranes since they interfere with their functions and with proteins inserted in membranes. PAHs can be mutagenic and carcinogenic in animals and humans (Mojiri et al., 2019; Patel et al., 2020).

In Chile, there are several coastal sites that are contaminated with residues of petroleum refineries and copper smelters rendering soils, sediments, and seawater contaminated with hydrocarbons and heavy metals, mainly copper and arsenic (Parra et al., 2015; Oyarzo-Miranda et al., 2020). In addition, oil spillages have occurred several times over the past 20 years increasing contamination in these sites, thus endangering the population health (Larsson, 2020). In these contaminated coastal zones, primary producers, such as marine macroalgae, are affected since it has been observed that brown macroalgae *Macrocystis pyrifera* and *Lessonia spicata* showed inhibited growth, germination, and spore settlement (Jara-Yañez et al., 2021; Meynard et al., 2021). Thus, it is important to find biotechnological tools that allow the remediation of seawater from hydrocarbons and heavy metals.

In animals and plants, hydrophobic compounds and xenobiotics, including hydrocarbons and PAHs, are metabolized cells *via* Phase I and Phase II detoxification reactions (Jancova et al., 2010). Phase I reactions tend to render the compounds more soluble in water by adding hydroxyl groups through the action of monooxygenases and dioxygenases. Phase II reactions transformed hydroxylated compounds in more soluble molecules by adding a molecule of glucose, glucuronic acid, glutathione, sulfate, or acetyl groups, and metabolized molecules are more easily excreted (Jancova et al., 2010). PAHs can be directly metabolized by different enzymes such as monooxygenases and dioxygenases that are present in bacteria, fungi, algae, and plants (Liu et al., 2017; Patel et al., 2020). In fungi, ANT is oxidized to anthraquinone and phthalic anhydride by dioxygenases, to formylbenzoic acid by epoxide hydrolases, to phthalic acid and muconic acid by dioxygenases, and then to acetyl-CoA and succinyl-CoA that enter the tricarboxylic acid (TCA) cycle to produce CO_2_ (Patel et al., 2020; Dell'Anno et al., 2021). For example, the fungus *Polyporus* sp. S133 isolated from petroleum-contaminated soil in Japan showed the latter catabolic pathway for ANT degradation (Hadibarata et al., 2012). In addition, the bacterium *Bacillus licheniformis* strain MTCC 5544 isolated in India utilized a similar degradation pathway for ANT metabolization (Swaatly et al., 2014) indicating that the anthraquinone/salicylic acid/phthalic acid pathway exists in fungi and bacteria. On the other hand, it has been shown that the halophilic bacterium *Martelella* sp. strain AD-3 isolated from petroleum-contaminated soil in China (Cui et al., 2014) and in *Bacillus fusiformis* isolated from refining wastewater sludge in China (Lin et al., 2010) can degrade PAHs involving the enzymes hydroxyphenylpyruvic acid dioxygenase (HPPDO) and homogentisic acid dioxygenase (HGDO). Thus, there are two main ANT degradation pathways in bacteria and fungi that can metabolize ANT involving mainly dioxygenases and monooxygenases.

Recently, it has been shown that the marine bacterium *Alcaligenes aquatilis* strain QD168, isolated in Quintero Bay, Valparaíso, Chile during oil spillage observed in 2014, metabolized several components of crude oil and its genome encode genes involved in seven catabolic pathways that allow the degradation of benzene, phenol, toluene, benzoic acid, catechol, salicylic acid, protocatechuic acid, hydroxyphenylpyruvic acid, gentisic acid, homogentisic acid, and many other aromatic compounds, but not ANT or PHE (Durán et al., 2019). In contrast, marine bacterium *A. aquatilis* strain B33N isolated in northern Tunisia was able to metabolize PHE, crude oil, and alkanes, indicating that this strain may present genes involved in PAHs and linear hydrocarbons degradation, but these genes were not identified (Mahjoubi et al., 2019). Thus, different strains of the same bacterium may present different metabolic pathways to degrade PAHs.

In terrestrial plants, it has been shown that *Arabidopsis thaliana* exposed to 0.25 mM of PHE for 21 days showed an increased expression of several enzymes such as 2-oxoglutarate Fe (II) dioxygenases (2-OGDOs), a flavonol synthase, auxin oxidase, naringenin-3 dioxygenase, gibberellin 3-oxidase, leucoanthocyanidin oxygenase, and others, and these enzymes are involved phytohormones and flavonoids hydroxylation (Weisman et al., 2010; Hernández-Vega et al., 2021). In fact, the cloned and purified enzymes flavonol synthase and auxin oxidase degraded PHE *in vitro* (Hernández-Vega et al., 2017, 2021). Regarding PAH metabolization in microalgae, the green microalga *Chlamydomonas rienhardtii* cultivated with benzo[a]anthracene at 10 mg ml^−1^ completely metabolized this PAH after 11 days, and this process involved the enzymes HGDO, an enzyme involved in aromatic tyrosine catabolism, carboxymethylenebutenolidase, an enzyme involved in dichlorobenzene degradation, and a ubiquinol oxidase involved in oxidation of ubiquinone (Luo et al., 2020).

In marine macroalgae, the green macroalgae *U. intestinalis, Chladophora glomerata*, and *Chara aspera* cultivated with 20 mg ml^−1^ of BaP metabolized 42–49% after 6 h, and this process involved peroxidases and O-diphenol oxidases (Kirso and Ihra, 1998). In contrast, the brown macroalgae, *Fucus vesiculosus* and *Chorda filum*, accumulate 89–99% of BaP but only metabolized 4% after 5 days (Kirso and Ihra, 1998). The brown macroalga *Laminaria japonica* cultivated with 0.1 mg ml^−1^ of PHE, and PYR showed degradation of 90% after 14 days (Wang and Zhao, 2007). Recently, it was shown that the green macroalga *Ulva lactuca* cultivated with 5 μM (1.26 mg ml^−1^) of ANT rapidly removed this PAH from the culture medium (6 h) and completely degraded it after 48 h (González et al., 2021). In addition, it was shown using specific inhibitors that monooxygenases and dioxygenases are involved in ANT metabolization in *U. lactuca* (González et al., 2021).

In this work, transcriptomic analyses were performed with total RNA of *U. lactuca* cultivated without ANT and with 5 μM of ANT for 24 h in order to identify overexpressed genes that may be involved ANT degradation. Three degradation pathways for PAHs and linear hydrocarbons were proposed based on overexpressed genes. The expression of transcripts was analyzed in the alga cultivated with 5 μM of ANT for 0–48 h using qRT-PCR, and ANT metabolites were analyzed by gas chromatography and mass spectrometry (GC–MS), allowing the validation of one pathway.

## Materials and methods

### Algal sampling

*Ulva lactuca* was collected in Cachagua (32° 24′S, 71°, 27′W), a non-polluted site of central Chile, transported in a cooler with ice, manually cleaned, and sonicated twice to remove epiphytic bacteria. The alga was maintained in artificial seawater prepared using 300 g of sea salts (Sigma-Aldrich, CA, USA) in 1 L of distilled water, at 14°C, with air bubbling, and a photoperiod of 14 h light and 10 h darkness.

### *In vitro* cultures

To prepare total RNA for transcriptomic analyses, the alga (1 g of fresh tissue [FT]) was cultivated in 30 ml of artificial seawater without ANT (control sample) and with 5 μM of ANT for 24 h (treated sample) at 14°C, with air bubbling and with a photoperiod of 14 h light and 10 h darkness, in duplicate.

To amplify transcripts encoding metabolizing enzymes by qRT-PCR, the alga (1 g of FT) was cultivated in artificial seawater with 5 μM of ANT for 0, 6, 12, 24, and 48 h at 14°C with air bubbling and with a photoperiod of 14 h light and 10 h darkness in triplicate.

To extract ANT metabolites, the alga (10 g of FT) was cultivated in 300 ml of artificial seawater with 5 μM of ANT for 0–48 h at 14°C with air bubbling and with a photoperiod of 14 h light and 10 h darkness, in triplicate.

### Total RNA extraction for transcriptomic analyses

Total RNA was extracted from *U. lactuca* (100 mg of FT) cultivated without ANT and with 5 μM ANT for 24 h using EZNA total RNA Kit (Omega Bio-Tek, GA, USA). Total RNA was sent to BGI Genomics Center (Shenzhen, China), paired ended libraries were prepared, and sequenced using a HiSeq Illumina 4000 instrument.

### Cleaning of the reads, transcriptome assembly, identification differentially expressed transcripts, and overexpressed transcripts

Reads obtained by Illumina (817,741,776). This represents billions of reads were visualized using FastQC program and subjected to quality control using PrinSeq and Trimmomatic programs. *De novo* transcriptome was assembled using Trinity software, and sequences were blasted using Blast X program and NR database. Cleaned reads (716,210,752), which represent 87.6% of initial reads were mapped against the *de novo* transcriptome using Bowtie2 software (Langmead and Salzberg, 2012), and they were counted using RSEM software (Li and Dewey, 2011). Differentially expressed transcripts (507,406) were identified using DESeq2 software (Love et al., 2014) at an FDR < 0.05 and Log2 fold of change of >2 and 4,419 upregulated transcripts, and 846 downregulated transcripts were detected. Completeness of the transcriptome was 88% using BUSCO software.

### Selection of overexpressed dioxygenases, monooxygenases, and other hydrocarbon metabolizing enzymes

The 4,419 overexpressed transcripts were analyzed, and those encoding monooxygenases and dioxygenases and other hydrocarbon metabolizing enzymes were curated by hand among the overexpressed transcripts, and those having different nucleotide sequences were selected.

### Quantification of overexpressed transcript levels by qRT-PCR

Total RNA was extracted from 50 mg of algal FT cultivated with 5 μM of ANT for 0–48 h using EZNA total RNA Kit (Omega Bio-Tek, GA, USA). Total RNA (2 μg) was used to synthesize cDNA using AffinityScript qPCR cDNA Synthesis Kit (Agilent, Santa Clara, CA, USA). Quantitative RT-PCR was performed using 50 ng of cDNA, 400 nM of each primer, and Ultrafast SYBR Green qPCR Kit (Agilent, Santa Clara, CA, USA) and Aria MX real-time PCR system (Agilent, Santa Clara, CA, USA). The amplification program consisted in an initial step of 30 s at 95°C and 40 cycles of 15 s at 95°C, 5 s at 58°C, and 30 s at 60°C. Primers were designed based on sequences of monooxygenases and dioxygenases and other metabolizing enzymes identified in transcriptomic analyses. Primer sequences are listed in Table 1. It is important to mention that transcript level of 18S rRNA did not change in control and treated samples.

**Table 1:** Primers used for qRT-PCR of ANT degradation enzymes genes.

**Gene**	**Primer sequences**	**Product size (bp)**
2-oxoglutarate-dependent dioxygenase	F - CGTGACTTCCAGCTCAGGTT R - CCTGATGCTGCCAGTAGGTC	115
Flavin-dependent monooxygenase	F- TGTGCTCAAACTTGGCTGGA R - TACCGGTGTGTATGATGCGG	104
Epoxide hydrolase	F - AGGGCTGGCTGAAGAAAGAC R - GGAAGTAGGTGCTCAGCGTT	129
4-hydroxyphenylpyruvic acid dioxygenase	F - TGAGTGTTCTGGCGCCTAAG R - ACAAAACGCAGCACGACATC	108
Homogentisic acid 1,2-dioxygenase	F - ATGGTGTTGCGGTGGAAGTA R - TTCGTGCCGTTCAAGTACGA	203
Alkane monooxygenase	F – TCGCGGTACAGGTTTAGCAG R - CATGTCCGATGACACCACCA	153
18S rRNA	F - GTGCACAAAATCCCGACTCT R - GCGATCCGTCGAGTTATCAT	148

### Anthracene metabolites extraction and analyses by GC–MS

To identify ANT metabolites, 10 g of the alga were cultivated with 5 μM of ANT for 0–48 h, in triplicate. Algae samples were lyophilized and homogenized in a mortar with a pestle at room temperature. In total, 300 ml of diethyl ether was added, and the mixture was sonicated for 30 min. The mixture was filtered using a 0.2-μm filter, the solvent was evaporated using rotatory evaporator, and the pellet was solubilized in 2 ml of cyclohexane.

A sample of 1 μl was injected into a GC–MS apparatus (PerkinElmer, Clarus 500, MA, USA) implemented with a capillary column of 30 m length (PerkinElmer Elite-5MS) having an inner diameter of 0.25 mm, and a thickness of 0.25 μm (PerkinElmer, Waltham, MA, USA). Helium was used as a carrier, at a flux of 1 ml min^−1^, and the injector temperature was 265°C in split-less mode. The temperature of the column was initially 40°C for 1 min, then 120°C at the rate of 25°C/min, then 160°C at the rate of 10°C/min, and finally 300°C at the rate of 5°C min^−1^. The detector temperature was at 280°C with a voltage of 1.5 kV. MS analysis was performed using an electron impact ionization of 70 eV in the m/z range of 100–400. The identification of ANT metabolites was performed using NIST 2015 Mass Spectra Library.

## Results and discussion

### ANT induced an increased level of transcripts encoding dioxygenases, monooxygenases, and other metabolizing enzymes

Transcriptomic analyses were performed using total RNA of the alga cultivated without ANT and with 5 μM of ANT for 24 h. Differentially expressed transcripts were selected, and overexpressed transcripts were identified. Among overexpressed transcripts, several dioxygenases, dienelactone hydrolases, monooxygenases, epoxide hydrolases, and others were identified (Table 2). Amino acid sequences of these enzymes showed not only similarity to green microalgae and plant enzymes but also to bacterial and fungal enzymes. These enzymes showed an average amino acid similarity of 60% and an average coverage in amino acids of 40% compared to enzymes different taxa (Table 2). Enzymes showing similarity to bacterial and fungal enzymes represent 35% of total identified enzymes (Table 2). It is possible that the genes encoding enzymes involved in ANT degradation having similarity with fungal and bacterial enzymes may have been acquired by the alga from marine bacteria and fungi by horizontal gene transfer (HGT). The latter has been initially detected in the genome of moss *Physcomitrella patens* exhibiting genes that are involved in the synthesis of auxin, polyamines, glutathione, cellulose, and starch, and in nitrogen assimilation, defense responses, and others, that have been have acquired by HGT form soil bacteria and fungi (Yue et al., 2012).

**Table 2:** Overexpressed genes potentially involve in anthracene and linear hydrocarbons degradation.

**ID transcript**	***Encoded enzymes***	**Log2 FC**	**Best blast hit**	**Classification**	**Similarity**	**Coverage**
TRINITY_DN31442_c0_g1_i6	Aromatic ring dioxygenase	22	*Paraburkholderia sp*.	Bacteria	40%	36%
TRINITY_DN1802_c0_g1_i8	2-oxoglutarate-Fe(II) dioxygenase	9	*Tistrella sp*.	Bacteria	76%	15%
TRINITY_DN4828_c0_g1_i9	2-oxoglutarate-Fe(II) dioxygenase	23	*Coccomyxa sp*.	Microalga	70%	66%
TRINITY_DN10280_c0_g1_i3	2-oxoglutarate-Fe(II) dioxygenase	11	*Micractinium conductrix*	Microalga	52%	48%
TRINITY_DN118124_c0_g1_i1	Dienelactone hydrolase	23	*Chloroflexi bacterium*	Bacteria	44%	42%
TRINITY_DN245293_c0_g1_i1	Dienelactone hydrolase	23	*Nannochloropsis gaditana*	Microalga	50%	51%
TRINITY_DN6370_c0_g1_i2	Dienelactone hydrolase	10	*Euryhalocaulis caribicus*	Bacteria	56%	32%
TRINITY_DN2194_c0_g1_i4	FAD-dependent monooxygenase	26	*Calothrix parasitica*	Cyanobacteria	52%	69%
TRINITY_DN9318_c0_g3_i2	FAD-dependent monooxygenase	11	*Leptolyngbya sp*.	Cyanobacteria	73%	13%
TRINITY_DN5586_c0_g1_i6	Cytochrome P450 monooxygenase	23	*Coccomyxa sp. Obi*	Microalga	46%	26%
TRINITY_DN35497_c0_g1_i1	Cytochrome P450 monooxygenase	23	*Coccomyxa sp. Obi*	Microalga	59%	69%
TRINITY_DN43794_c2_g2_i2	Cytochrome P450 monooxygenase	23	*Coccomyxa sp. Obi*	Microalga	53%	26%
TRINITY_DN241019_c0_g1_i1	Cytochrome P450 monooxygenase	23	*Chondrus crispus*.	Alga	55%	83%
TRINITY_DN16330_c0_g1_i19	Epoxide hydrolase	23	*Corchorus olitorius*	Plant	62%	8%
TRINITY_DN2760_c0_g1_i8	Epoxide hydrolase	24	*Dunaliella salina*	Microalga	62%	19%
TRINITY_DN813_c0_g1_i4	4-hydroxyphenyl pyruvic dioxygenase	26	*Coccomyxa sp*	Microalga	74%	39%
TRINITY_DN719_c0_g1_i8	Homogentisic acid dioxygenase	24	*Vitis vinifera*	Plant	59%	61%
TRINITY_DN719_c0_g1_i13	Homogentisic acid dioxygenase	11	*Auxenochlorella protothecoides*	Microalga	69%	42%
TRINITY_DN6348_c0_g1_i1	Alkane monooxygenase (alkB)	23	*Chryseobacterium sp*.	Bacteria	44%	32%
TRINITY_DN337_c0_g1_i1	Alcohol dehydrogenase	12	*Corallococcus terminator*	Bacteria	70%	48%
TRINITY_DN47079_c0_g1_i1	Alcohol dehydrogenase	25	*Gracilaria domingensis*	Alga	64%	74%
TRINITY_DN10754_c0_g2_i2	Aldehyde dehydrogenase	10	*Micractinium conductrix*	Microalga	56%	30%
TRINITY_DN1524_c0_g1_i9	Aldehyde dehydrogenase	25	*Nocardia cyriacigeorgica*	Fungus	40%	13%
TRINITY_DN5678_c0_g1_i5	Aldehyde dehydrogenase	24	*Coccomyxa sp. Obi*	Microalga	68%	36%
TRINITY_DN14524_c0_g1_i1	Alkene reductase	23	*Rivularia sp*.	Cyanobacteria	66%	78%
TRINITY_DN3061_c0_g3_i1	Alkene reductase	23	*Chlorella sorokiniana*	Microalga	74%	23%

**Figure 1:**
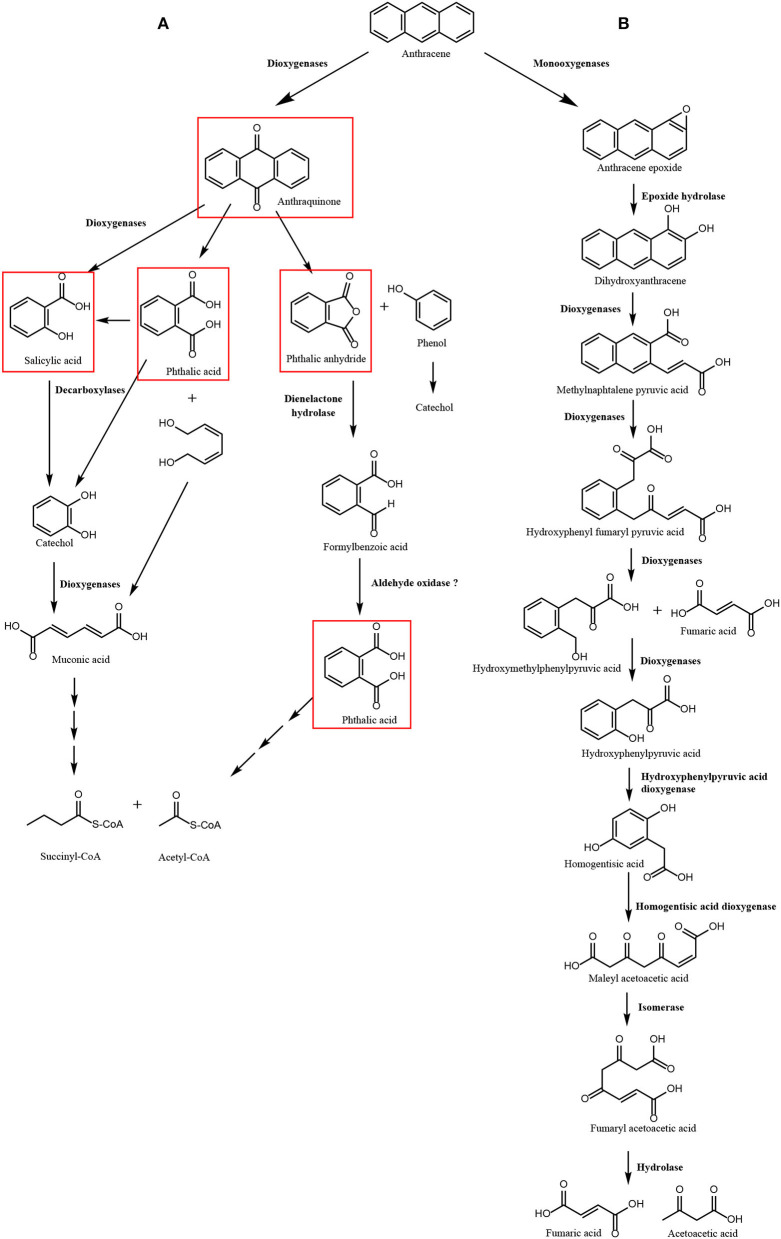
Scheme of pathways of anthracene (ANT) degradation in *Ulva lactuca*. Pathway 1 involves 2-oxoglutarate Fe (II) dioxygenases, aromatic ring dioxygenase and dienelactone hydrolase producing anthraquinone, phthalic anhydride, salicylic acid, and phthalic acid as ANT metabolites **(A)**. Pathway 2 that involves monooxygenases, epoxide hydrolase, 2-oxoglutarate dioxygenases, hydroxyphenylpyruvic dioxygenases, and homogentisic acid dioxygenases producing 1,2-dihydroxyanthracene, hydroxyphenylpyruvic acid, and homogentisic acid as ANT metabolites **(B)**. Metabolites detected by GC–MS analyses are framed in red.

In particular, three 2-OGDOs, one aromatic ring dioxygenase, and three dienelactone hydrolases were detected. These dioxygenases may involve the production of anthraquinone, phthalic anhydride, salicylic acid, and phthalic acid leading to the production of acetyl-CoA and succinyl-CoA that may enter in the TCA cycle to produce CO_2_. The latter enzymes may constitute the first pathway of ANT degradation (Figure 1A). In addition, two flavin adenine dinucleotide (FAD)-dependent monooxygenases, four cytP450 monooxygenases, two epoxide hydrolases, one 4-HPPDO, and two 2-HGDOs were identified (Table 2). These enzymes may be involved in the production of 1,2-dihydroxyanthracene, hydroxyphenylpyruvic acid, homogentisic acid, and maleylacetoacetic acid that will be converted into to fumaroylacetoacetic acid and to fumaric acid and acetoacetic acid that may enter the TCA cycle to produce CO_2_. The latter enzymes may constitute the second pathway of ANT degradation (Figure 1B). Thus, *U. lactuca* may present two main metabolic pathways of ANT degradation producing metabolites that may finally enter the TCA cycle to be converted into CO_2_.

The first pathway of ANT degradation in *U. lactuca* involving anthraquinone and phthalic acid was initially described in the fungus *Polyporus* sp. S133 isolated from petroleum-contaminated soil in Japan (Hadibarata et al., 2012), the bacterium *B. licheniformis* isolated from marine sediments in India (Swaathy et al., 2014), and the marine bacterium *A. aquatilis* strain B33N isolated in northern Tunisia (Mahjoubi et al., 2019). The second pathway involving production of hydroxyphenylpyruvic acid and homogentisic acid has been partially described in the halophilic bacterium *Martelella* sp. strain AD-3 isolated from petroleum-contaminated soil in China (Cui et al., 2014) and in the bacterium *B. fusiformis* isolated from refining wastewater sludge in China (Lin et al., 2010). Thus, mechanisms for ANT degradation in *U. lactuca* may show similarity to those found in fungi and bacteria, reinforcing the idea that some metabolizing enzymes involved in ANT degradation may have been acquired by *U. lactuca* from marine bacteria and fungi by HGT.

In contrast, algae do not display efficient mechanisms to metabolize PAHs. In this sense, it has been shown that the green macroalgae *U. intestinalis, Chladophora glomerata*, and *Chara aspera* cultivated with BaP metabolized only 42–49% after 5 days, and the brown macroalgae *Fucus vesiculosus* and *Chorda filum* metabolized only 4% after 5 days (Kirso and Ihra, 1998). In addition, the brown macroalga *Laminaria japonica* cultivated with PHE and PYR showed degradation of 90% after 14 days (Wang and Zhao, 2007). Furthermore, the green microalga *C. rienhardtii* completely metabolized benzo[a]anthracene after 11 days (Luo et al., 2020). In contrast, *U. lactuca* completely degraded ANT at 48 h of culture, indicating that this green macroalga display more efficient mechanisms than other macroalgae and microalgae for ANT degradation, which may be explained by the acquisition of genes of bacterial and fungal origin by HGT.

### ANT induced the expression of enzymes that may metabolize linear hydrocarbons

**Figure 2:**
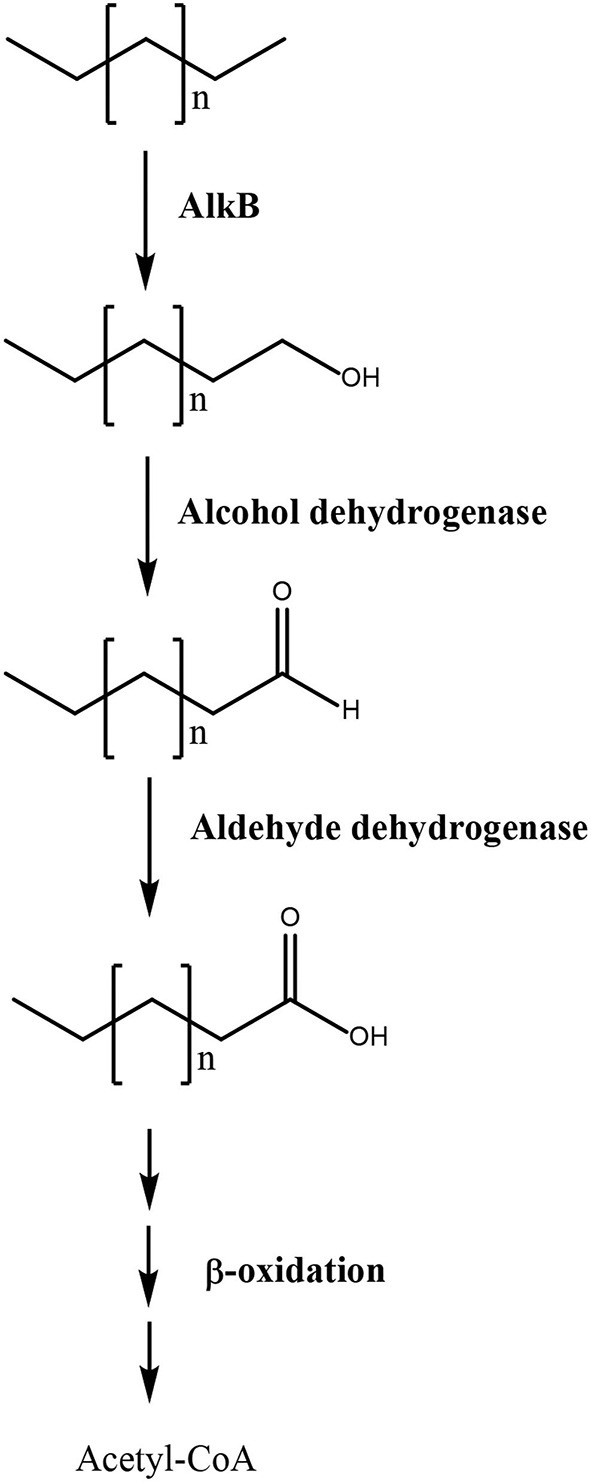
Scheme of linear hydrocarbons degradation in *Ulva lactuca*. Pathway 3 involves alkane monooxygenase (alkB), alcohol dehydrogenase, and aldehyde dehydrogenase that oxidize the carboxy terminal methyl group of alkanes to a hydroxyl, aldehyde, and carboxylic acid group, thus producing fatty acid that are metabolized by β-oxidation pathway to produce acetyl-CoA that enter the TCA cycle.

In addition, an alkane monooxygenase (alkB), two alcohol dehydrogenases, three aldehyde dehydrogenases, and two alkene reductases were identified among overexpressed transcripts. The first three enzymes may oxidize the carboxy terminal methyl group of linear hydrocarbons (alkanes) into an alcohol, that is further oxidized to an aldehyde and finally to a carboxylic acid, thus transforming alkanes into fatty acids that may be further oxidized to acetyl-CoA and enter the TCA cycle to be degraded into CO_2_ (Figure 2). The alkene reductase transforms alkenes into alkanes that are further metabolized through this pathway leading to fatty acids. In bacteria, it has been shown that the degradation of alkanes requires key enzymes such as alkB monooxygenase and cytP450 monooxygenases (Sabirova et al., 2006; Yakimov et al., 2007; Austin and Groves, 2011). Interestingly, the alkB monooxygenase in *U. lactuca* showed similarity to the enzyme of the bacterium *Chryseobacterium* sp., suggesting that this key gene for alkane degradation may have been transferred from a marine bacterium to the alga probably by HGT. On the other hand, it has been shown that *U. lactuca* collected in Brazil can degrade 1% of Brazilian gasoline that is constituted by 35.1% ethanol, 9.7% paraffinic hydrocarbons (alkanes), 18.6% of hydroparaffinic, 15.6% of PAHs, 10.9% naphthenic hydrocarbons, 6.4% olefinic hydrocarbons (alkenes), and 3.7% of unidentified hydrocarbons in 24 h (Kokovicz-Pilatti et al., 2016). The latter indicates that Brazilian *U. lactuca* can metabolize different types of hydrocarbons, but the genes and enzymes involved in gasoline degradation were not identified. In addition, the fact that Chilean *U. lactuca* can degrade linear hydrocarbons has not been experimentally demonstrated, but these experiments will be performed in the future.

### Quantification of transcripts encoding enzymes that may be involved ANT and lineal hydrocarbon degradation

**Figure 3:**
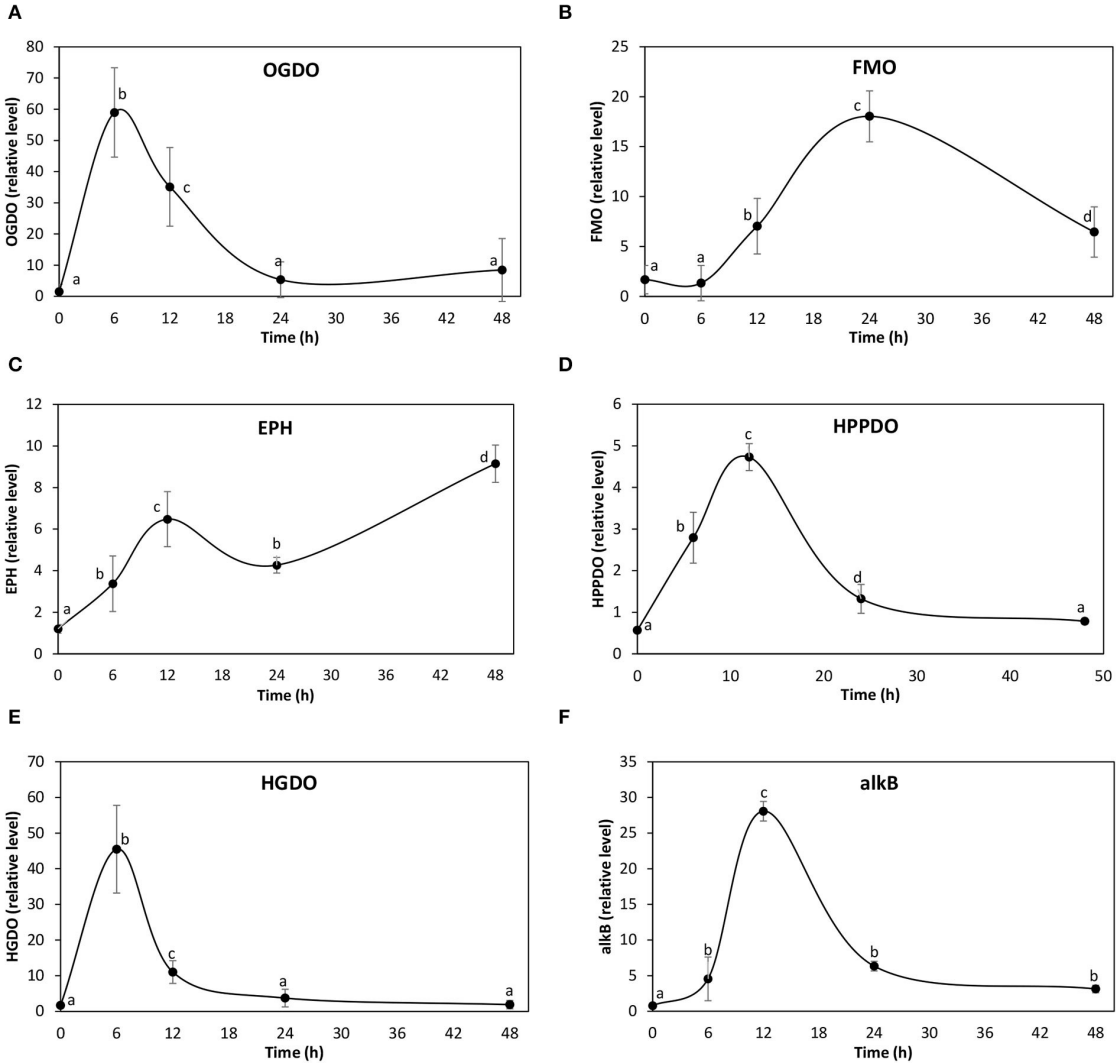
Relative level of transcripts encoding 2-oxoglutarate Fe (II) dioxygenases **(A)**, FAD-dependent monooxygenases **(B)**, epoxide hydrolases **(C)**, hydroxyphenylpyruvic acid dioxygenase (HPPDO) **(D)**, homogentisic acid dioxygenase **(E)**, and alkane monooxygenase (alkB) **(F)** in *U. lactuca* cultivated with 5 μM ANT for 0–48 h. The relative level of transcripts is expressed 2^−ΔΔCt^.

In order to verify the overexpression of the identified transcripts, the level of transcripts of three 2-OGDOs, two FAD-dependent monooxygenases, two epoxide hydrolase, one HPPDO, two HGDOs, and one alkane dioxygenase were analyzed in the alga cultivated with 5 μM of ANT for 0–48 h. The level of transcripts encoding the 2-OGDOs increased in response to ANT showing a maximal level at 6 h, and 39 times of increase, and then decreased reaching control level at 24 h of culture (Figure 3A). Transcripts encoding the FAD-dependent monooxygenases increased showing a maximal level at 24 h, and 11 times of increase, and then decreased to 6 times of increase at 48 h (Figure 3B). Transcripts encoding the epoxide hydrolases increased showing a maximal level at 12 h, and 5 times of increase, decreased to 4 times of increase at 24 h, and then increased reaching 9 times of increase at 48 h of culture (Figure 3C). Transcripts encoding the 4-HPPDO increased showing a maximal level at 12 h of culture, and 8 times of increase, and then decreased to control level at 48 h (Figure 3D). Transcripts encoding the 2-HGDO increased showing a maximal level at 6 h, and 27 times of increase, and then decreased to control level at 24 h of culture (Figure 3E). Transcripts encoding alkB monooxygenase increased showing maximal level at 12 h culture, and 36 times of increase, and then decreased to control level at 24 h of culture (Figure 3F). Thus, the enzymes that showed an early increase at 6 h of culture were 2-OGDOs and alkB monooxygenase. In addition, the enzymes showing higher expression were 2-OGDOs, alkB monoxygenase, and HGDOs, suggesting that they are key enzymes involved in polycyclic aromatic and linear hydrocarbons degradation.

It has been shown that 2-OGDO, flavonol synthase, and auxin oxidases increased in response to PHE after 21 days in A. *thaliana* (Weisman et al., 2010; Hernández-Vega et al., 2021). Interestingly, the cloned flavonol synthase and auxin oxidase were able to degrade PAHs *in vitro* (Weisman et al., 2010; Hernández-Vega et al., 2021). All these enzymes hydroxylate phytohormones and flavonoids in plants (Kawal et al., 2014). Here, it was shown that 2-OGDOs are overexpressed in response to ANT and with high level indicating that this enzyme may participate in the degradation of ANT in *U. lactuca*. However, these 2-OGDOs do not participate in the hydroxylation of phytohormones such as gibberellins since these phytohormones may not be present in *U. lactuca*, as it has been shown in *U. mutabilis* (De Clerck et al., 2018). In addition, the genome of another Ulvophyceae, *U. compressa*, was recently sequenced, and genes encoding enzymes involved in the synthesis of auxin, gibberellins, and cytokinins were not identified (Osorio et al., 2022). Thus, probably 2-OGDOs are not required for phytohormone activation synthesis in *U. lactuca*.

### ANT inhibited the expression of proteins and enzymes involved in protein synthesis and degradation, gene expression, signal transduction, and others

Transcriptomic analyses allowed the identification of 846 downregulated transcripts and 254 (30%) did not show similarity to known genes in NCBI database. Transcripts that were downregulated encode proteins and enzymes involved in protein synthesis and degradation, regulation of gene expression, signal transduction, ion channels, and metabolite transporters. Transcripts encoding proteins involved in protein synthesis were ribosomal proteins S8, S12, S22, S30, L9, L40, and L47; translation elongation factors EF1a, EF2b, EF2, and EF4; three peptidyl-prolyl isomerases and chaperones CBLP, DNA J (Hsp40), and GrpE; and tRNA-modifying enzymes such as tRNA methyl transferase, tRNA-dihydrouridine synthase, tRNA adenosine deaminase, and others. Transcripts encoding proteins involved in protein degradation were ubiquitin-conjugating enzymes E2 and E3, ubiquitin protease, ubiquitin carboxy terminal hydrolase, RING finger protein, cysteine protease, metacaspase-1, ATP-dependent Zn protease, Zn-dependent oligopeptidase, Fanconi-associated protease, IPAL protease, aminopeptidase N, and others.

Proteins involved in the regulation gene expression were transcription factors MYB44, TFY, and AP-2 ethylene-responsive factor; DNA repair helicase, DNA repair subunit of transcription actor TFIIH, DNA repair enzyme RAD50, ATP-dependent DNA helicase, ATP-dependent RNA helicase, and DNA topoisomerase 6; two GNAT acetyltransferases; and two histone deacetylases, histone demethylase, ADP-ribosylation factor 2, SAM-dependent methyltransferase, and others. Proteins involved in signal transduction were eight serine–threonine protein kinases, three CaMK, AGC family protein kinase, histidine kinase, phosphatidylinositol 3,4 kinase, inositol phosphate epimerase, phosphatidylinositol diphosphatase, phospholipase A2, phospholipase A2 activator, phosphodiesterase, and others. Transcripts encoding ion channels were two chloride channels and a calcium channel ATPase of endoplasmic reticulum. Transcripts that encode metabolite transporters were ADP/ATP transporter, phosphoenolpyruvate transporter, proline transporter, serine transporter, cholesterol transporter, two choline transporters, carnitine transporter, phospholipid transporter, two Na^+^/Cl^−^ transporters of GABA, Na^+^/Ca^2+^ exchanger, Na^+^/H^+^ antiporter, and others.

### Detection of ANT metabolites

**Figure 4:**
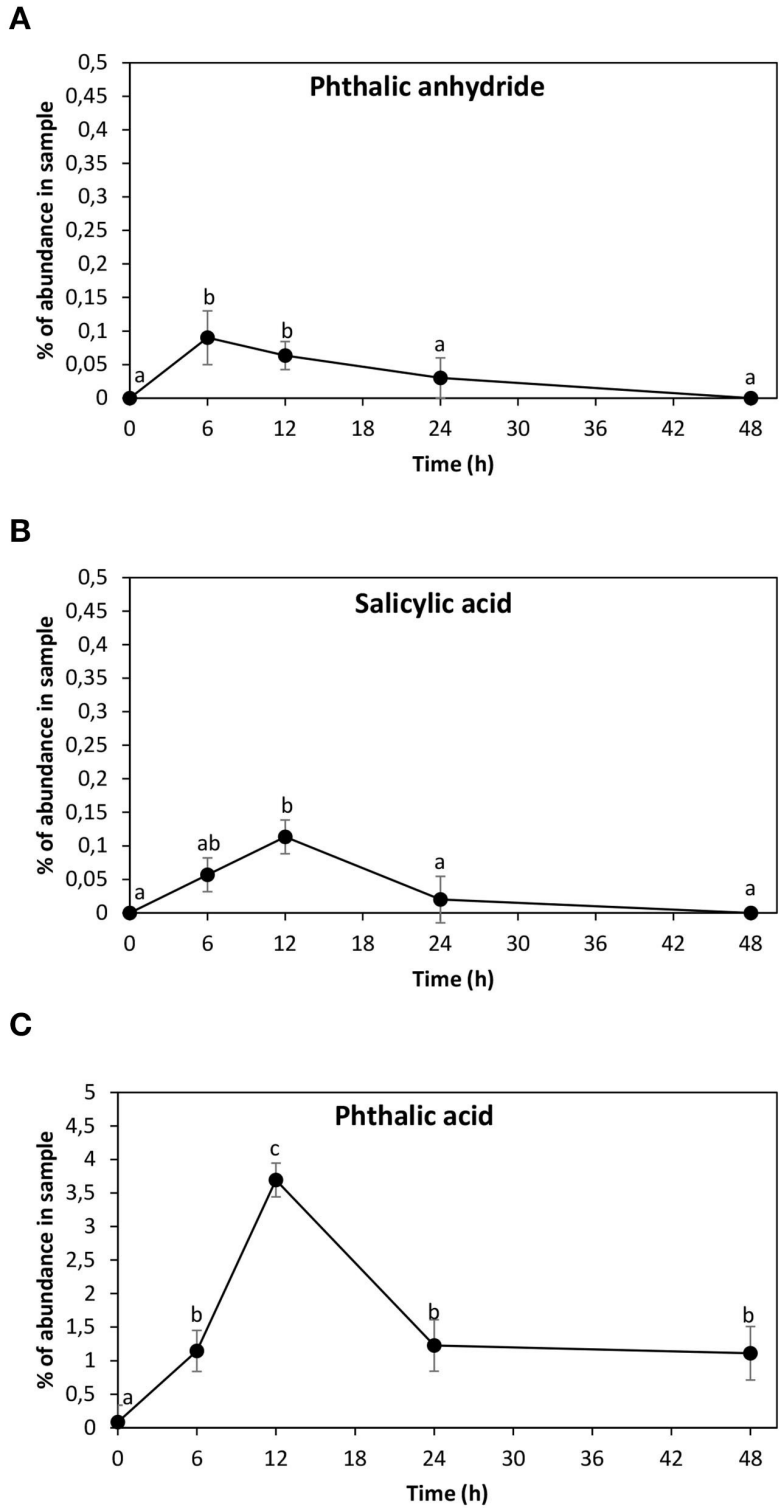
Level of anthracene (ANT) metabolites corresponding to phthalic anhydride **(A)**, salicylic acid **(B)**, and phthalic acid **(C)**. The level of each metabolite is expressed in percentage of abundance in the sample injected into GC–MS column. Symbols represent the mean value of three independent experiments. Different letters indicate significant differences among mean values (±SD) (*p* < 0.05).

The ANT metabolites were analyzed in the alga cultivated with 5 μM of ANT for 0–48 h using GC–MS technique. Three ANT metabolites were detected corresponding to phthalic anhydride with an RT = 41.87 min, m/z coefficients of 166 (parent ion) and 148, 104, 76, and 50 (Supplementary Figure 1A); salicylic acid showing an RT = 22.4 min, m/z coefficients of 138 (parent ion), 120, 92, and 64 (Supplementary Figure 1B), and phthalic acid with an RT = 34.8 min and m/z coefficients of 148 (parent ion), 104, 76, and 50 (Supplementary Figure 1C). In addition, a previous analysis performed in the alga cultivated with 5 μM of ANT for 24 h showed the metabolite anthraquinone (Supplementary Figure 1D). The finding of anthraquinone, phthalic anhydride, salicylic acid, and phthalic acid validate pathway 1, and these metabolites are framed in red in Figure 1. The level of phthalic anhydride increased reaching a maximal level at 6 h and then decreased until 48 h (Figure 4A). The level of salicylic acid increased to reach a maximal level at 12 h and then decreased to control level until 24 h (Figure 4B). The level of phthalic acid increased reaching a maximal level at 12 h and then decreased at 24 h and remained unchanged until 48 h (Figure 4C). The level of phthalic acid was 38 times higher than those of phthalic anhydride and salicylic acid, and the accumulation of this metabolite may be explained by the fast oxidation of ANT to phthalic acid and a slower oxidation to acetyl-CoA and succinyl-CoA (Figure 1).

## Conclusion

The marine alga *U. lactuca* (Ulvophyceae) can completely metabolize 5 μM of ANT after 48 h. Transcriptomes prepared with total RNA of the alga cultivated with 5 μM of ANT for 24 h showed an increased expression of one aromatic ring dioxygenases, three 2-OGDOs, and three dienelactone hydrolases. These enzymes may account to ANT oxidation to anthraquinone, salicylic acid, and phthalic acid and then to succinyl-CoA and acetyl-CoA that can enter the TCA cycle to produce CO_2_ (pathway 1). In addition, an increased expression of two FAD-dependent monooxygenases, two epoxide hydrolases, one HPPDO, and two HGDOs were detected. These enzymes may account for ANT degradation to 1,2-dihydroxyanthracene, hydroxyphenylpyruvic acid, homogentisic acid, maleylacetoacetic acid, and to fumaric acid and acetoacetic acid that can enter the TCA cycle (pathway 2). Furthermore, ANT metabolites, such as anthraquinone, phthalic anhydride, salicylic acid, and phthalic acid, were detected which validate pathway 1. Furthermore, an increased expression of alkB monooxygenase, alcohol dehydrogenase, and aldehyde dioxygenases were detected. These enzymes may account for alkane oxidation into fatty acids that can degrade through β-oxidation pathway to produce acetyl-CoA that enters the TCA cycle (pathway 3). Thus, there are three potential mechanisms for ANT and linear hydrocarbons degradation in *U. lactuca*.

## Data availability statement

The datasets presented in this study can be found in online repositories. The names of the repository/repositories and accession number(s) can be found below: https://www.ncbi.nlm.nih.gov/, PRJNA842934.

## Author contributions

AG extracted total RNA for transcriptomic analyses. AG, SR, PM, and MS did algal cultures, total RNA extraction, and qRT-PCR. HO and DL did the bioinformatic analyses. MG-C and RS did GC–MS analyses. DL and EC-N participated in discussion of results. AM wrote the manuscript.

## Funding

This work was financed by ANID Fondecyt de Iniciación 11180189, ANID+PAI Concurso Nacional de Inserción de Capital Humano Avanzado en la Academia convocatoria 2017, PAI79170105, ANID Fondecyt de Iniciación 11200329, CONICYT Fondequip/GC MS/MS EQM 150084, Financiamiento Basal AFB180001, CEDENNA, and Dicyt-USACH.

## Conflict of interest

The authors declare that the research was conducted in the absence of any commercial or financial relationships that could be construed as a potential conflict of interest.

## Publisher's note

All claims expressed in this article are solely those of the authors and do not necessarily represent those of their affiliated organizations, or those of the publisher, the editors and the reviewers. Any product that may be evaluated in this article, or claim that may be made by its manufacturer, is not guaranteed or endorsed by the publisher.
