# Supplementary material for: Transcriptomic analyses reveal increased expression of dioxygenases, monooxygenases, and other metabolizing enzymes involved in anthracene degradation in the marine alga Ulva lactuca
Source: Front Plant Sci. 2022 Sep 20;13:955601. doi: 10.3389/fpls.2022.955601 (PMC9530894; doi:10.3389/fpls.2022.955601)
Supplement: Supplementary file 1 [file Data_Sheet_1.PDF]

A

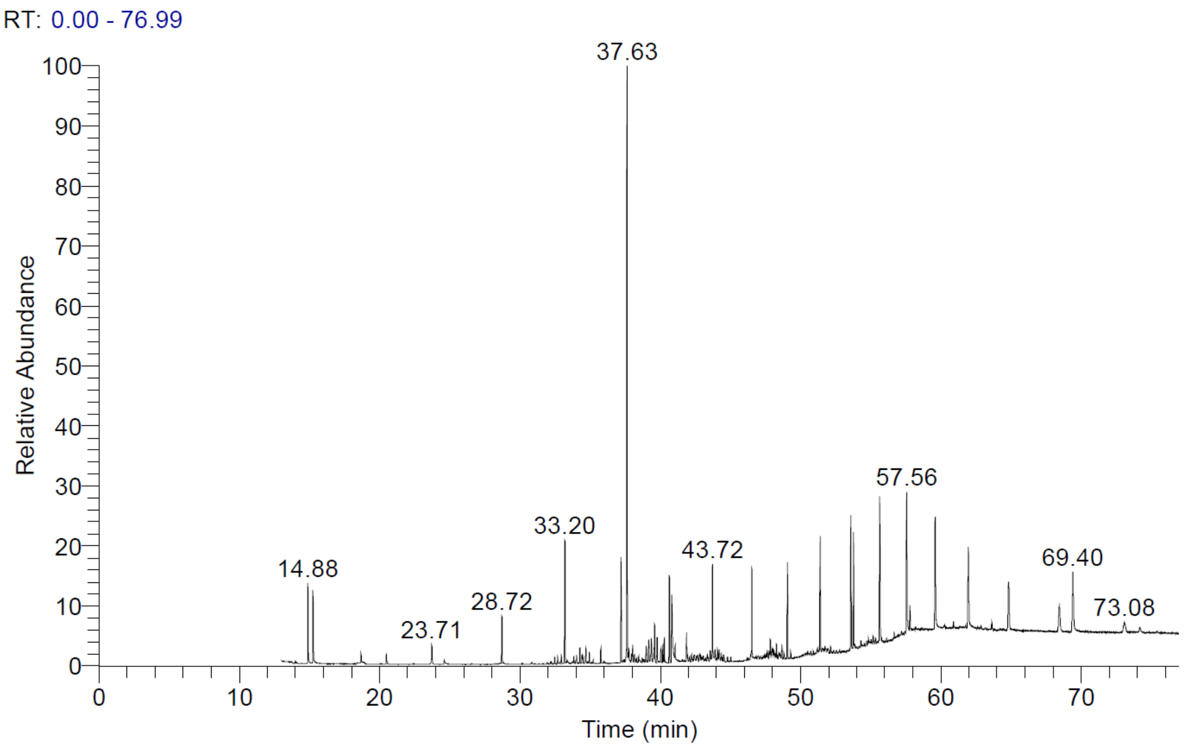

RT: 34,80
Phthalic anhydride (2-Benzofuran-1,3-dione)
m/z: 148, 104, 76, 50

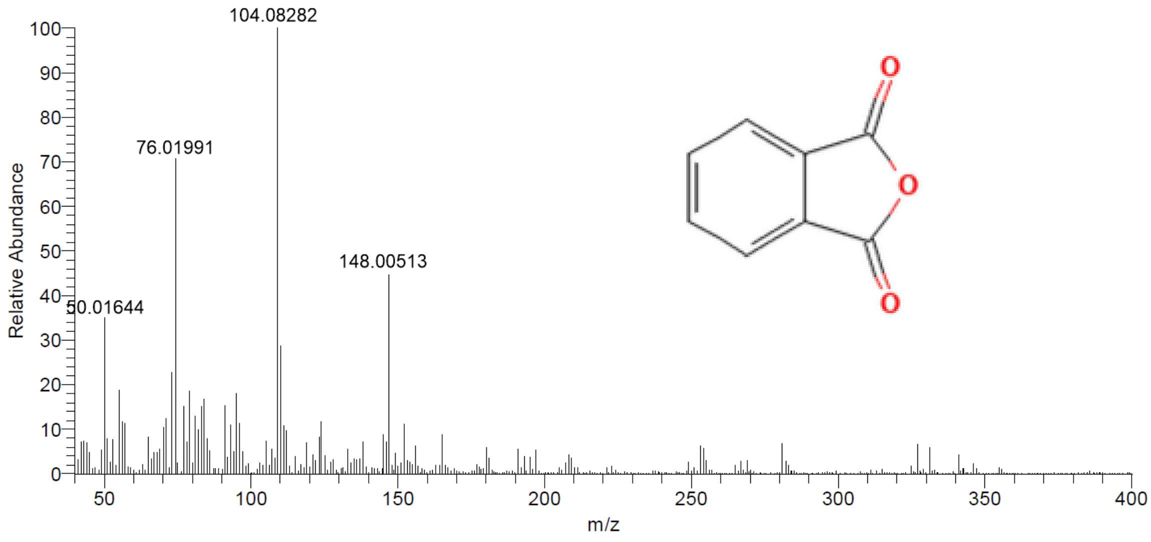

B

RT: 22.40
Salicylic acid (2-Hydroxybenzoic acid)
m/z: 138, 120, 92, 64

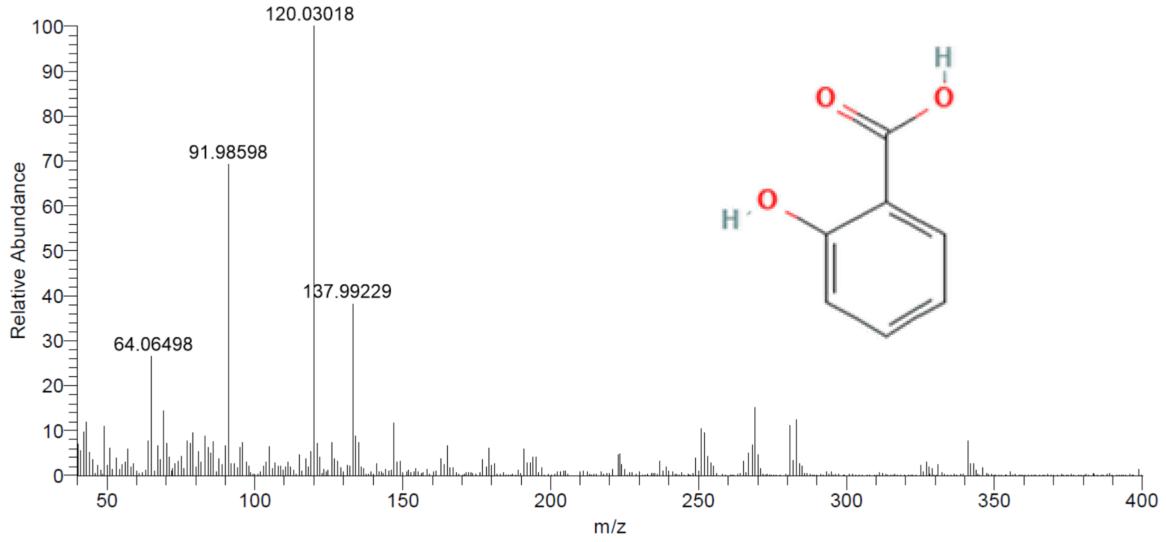

C

RT: 41.87
Phthalic acid (1,2-benzenedicarboxylic acid)
m/z: 166, 148, 104, 76, 50

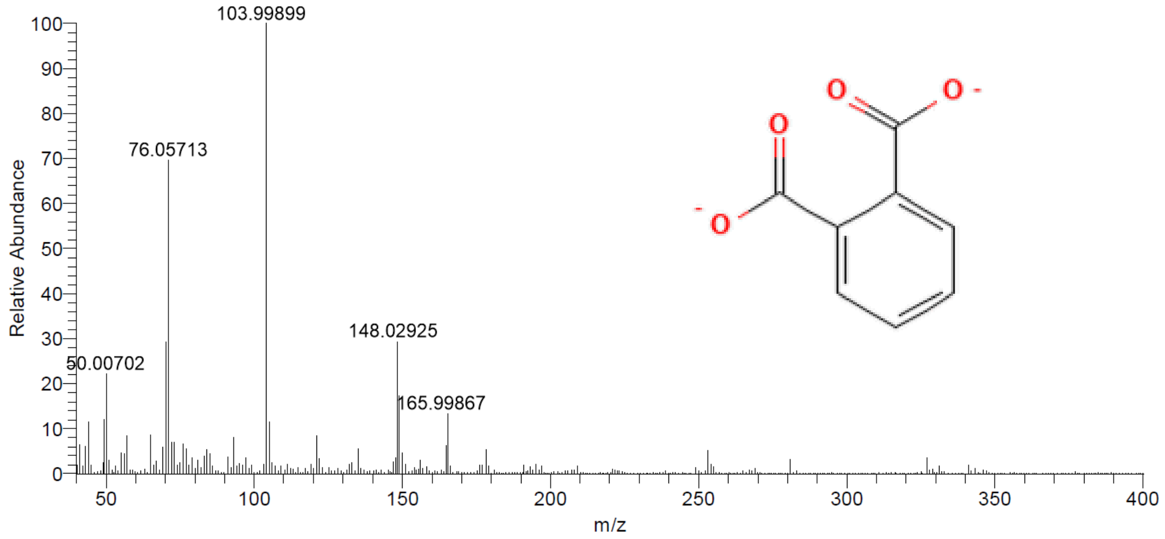

D

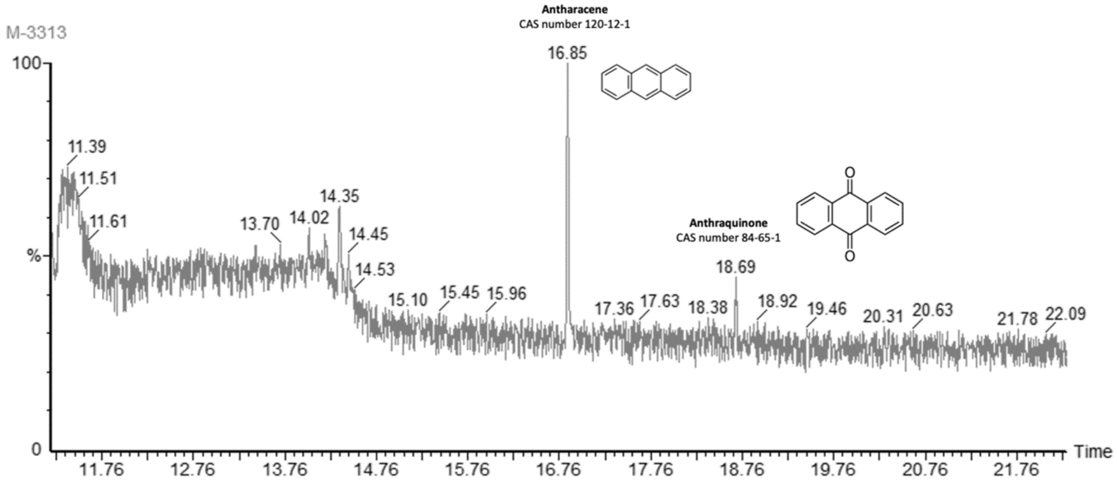

RT: 18.69
Anthraquinone (9,10-anthracenedione)
m/z: 208, 180, 152, 76

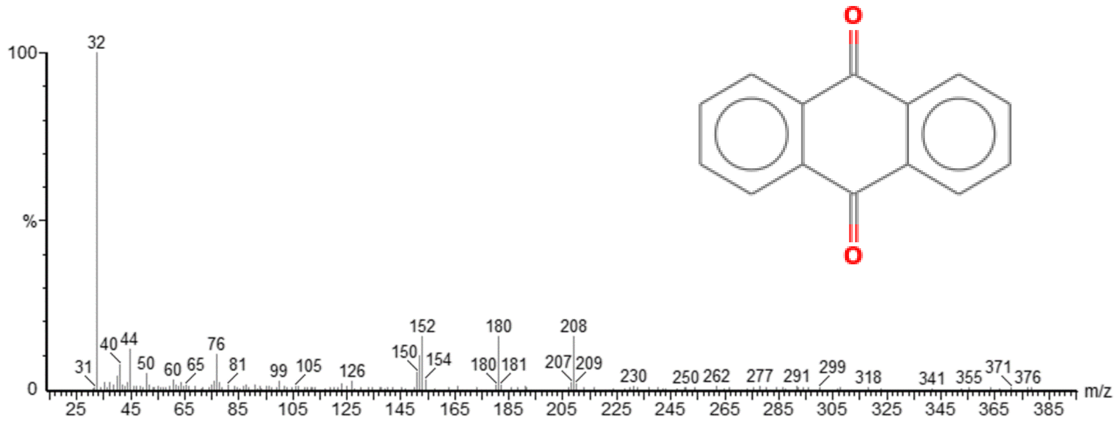

Fig. S1
